# Laparoscopic Anterior Gastropexy with a Technical Modification Using the Round Ligament for Internal Hernia of the Stomach after Sigmoid Colostomy: A Case Report

**DOI:** 10.70352/scrj.cr.26-0393

**Published:** 2026-10-02

**Authors:** Takahiro Saito, Susumu Fukahori, Koji Hontani, Takashi Kuroda, Sato Nishida, Tomoya Matsuda, Taku Maejima, Seiichi Minami, Masahiro Hagiwara, Satoshi Hirano

**Affiliations:** 1 Department of Surgery, Sapporo Higashi Tokusyukai Hospital, Sapporo, Hokkaido, Japan; 2 Department of Gastroenterological Surgery II, Hokkaido University Faculty of Medicine, Sapporo, Hokkaido, Japan

**Keywords:** internal hernia, colostomy, gastropexy, round ligament, laparoscopy, Hartmann’s procedure, gastric incarceration, colorectal cancer

## Abstract

**INTRODUCTION:**

Internal hernia involving the stomach associated with a sigmoid colostomy is extremely rare. The hernia defect is typically located between the colostomy limb and the left abdominal wall. Because only a few cases have been reported, no standardized management strategy has been established, particularly in patients in whom immediate colostomy closure is not appropriate.

**CASE PRESENTATION:**

A woman in her 70s underwent a laparoscopic Hartmann’s procedure with sigmoid colostomy for obstructive sigmoid colon cancer with para-aortic lymph node metastasis. On POD 9, the patient developed acute abdominal distension and vomiting. CT revealed marked gastric dilatation due to incarceration of the stomach in the space between the colostomy limb and the left lateral abdominal wall. Nasogastric decompression relieved the incarceration, and elective laparoscopic surgery was performed 4 days later to prevent recurrence. Because the remaining sigmoid colon was short and colostomy reconstruction would have required mobilization of the splenic flexure, anterior gastropexy was performed using a technical modification in which the prepyloric antrum was fixed to the round ligament of the liver, while the gastric body was fixed to the upper abdominal wall using nonabsorbable barbed sutures. The postoperative course was uneventful. The patient subsequently received systemic chemotherapy, followed by para-aortic lymph node dissection, during which no adhesiolysis involving the stomach or gastropexy site was required. No colostomy revision, fixation of the colostomy limb, or closure of the lateral defect was performed because staged colostomy closure was planned. Colostomy closure was subsequently recommended, but the patient declined additional surgery. No recurrence was observed during 24 months of follow-up.

**CONCLUSIONS:**

Internal hernia involving the stomach after sigmoid colostomy is extremely rare, and its optimal management remains unclear. Laparoscopic anterior gastropexy with a technical modification using the round ligament may be a feasible and minimally invasive option in selected patients, potentially maintaining physiological gastroduodenal alignment and avoiding excessive angulation of the gastric outlet.

## INTRODUCTION

Internal hernia involving the stomach after sigmoid colostomy is extremely rare. The hernia defect typically forms between the elevated colostomy limb and the left lateral abdominal wall, allowing the stomach to herniate into this potential space. To date, only a few such cases have been reported.^1–4)^ Although several surgical approaches have been described, including colostomy reconstruction and fixation procedures, no standardized management strategy has been established, particularly for patients in whom immediate colostomy closure is not appropriate. We report a rare case of laparoscopic anterior gastropexy with a technical modification using the round ligament of the liver as the cranial anchoring point, with the aim of preventing recurrent gastric incarceration while maintaining physiological gastroduodenal alignment and avoiding excessive angulation of the gastric outlet.

## CASE PRESENTATION

A woman in her 70s presented with hematochezia. Further evaluation revealed an obstructive sigmoid colon cancer with para-aortic lymph node metastasis. Considering the potential risk of perforation and the limitations of systemic chemotherapy associated with colonic stenting, primary tumor resection was prioritized. She underwent a laparoscopic Hartmann’s procedure with a sigmoid colostomy in the left lower abdomen. The patient’s immediate postoperative course was uneventful.

On POD 9, the patient developed acute abdominal distension and vomiting. CT revealed marked gastric dilatation caused by incarceration of the stomach in the space between the colostomy limb and the left lateral abdominal wall. Coronal images revealed a dilated stomach wrapped around the colostomy limb (**Fig. 1A**, **1B**). Because she was hemodynamically stable and showed no signs of ischemia, nasogastric decompression was performed, which resolved the incarceration. To prevent recurrence, elective laparoscopic surgery was scheduled 4 days later.

**Fig. 1:**
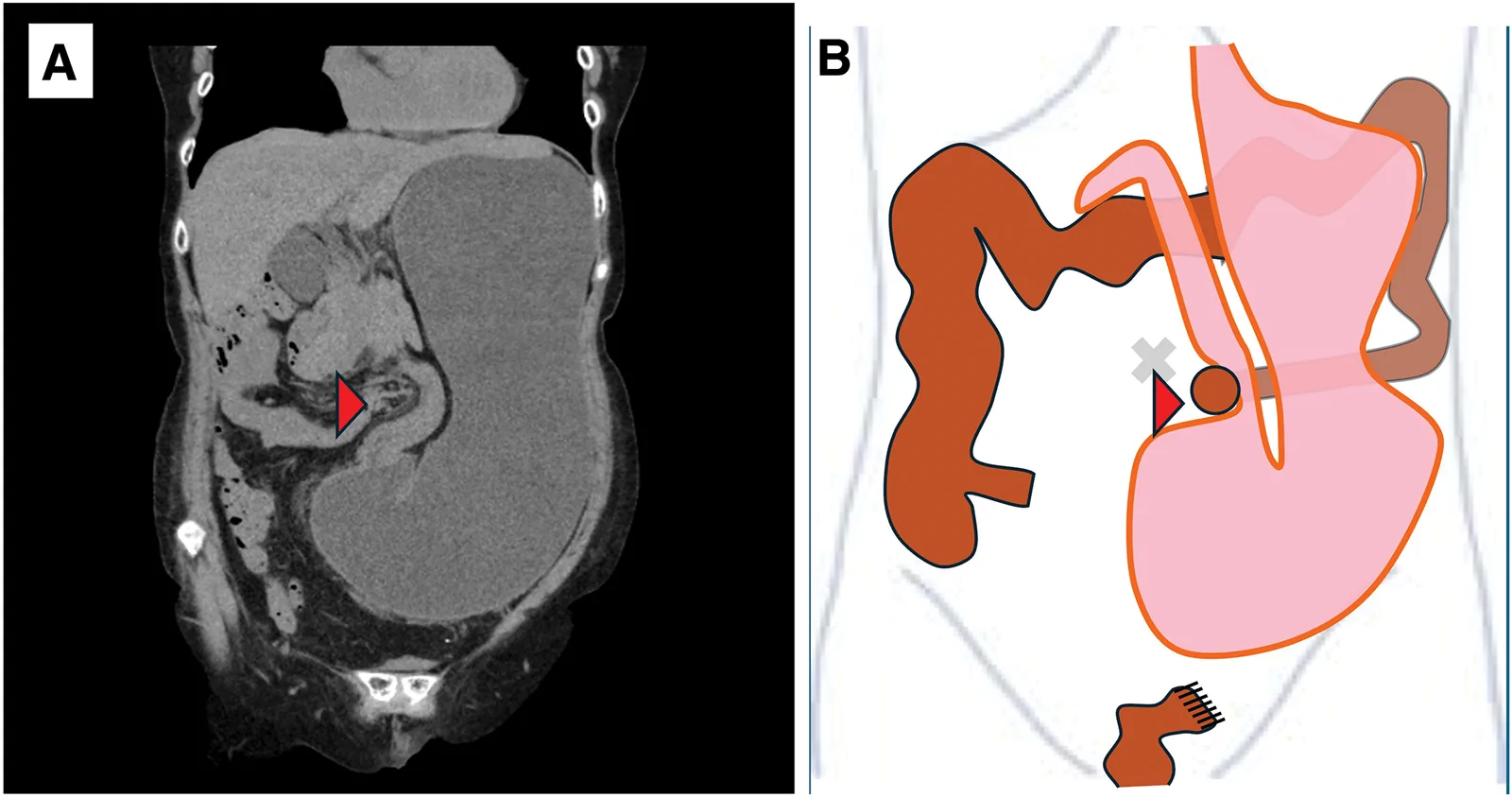
Preoperative imaging findings. (**A**) Coronal CT image. The triangle indicates the stoma. A markedly dilated stomach is seen wrapped around the colostomy limb. (**B**) Schematic illustration. The triangle indicates the stoma. The stomach is incarcerated in the space between the colostomy limb and the left lateral abdominal wall.

A 12 mm port was placed at the umbilicus, and two 5 mm ports were inserted in the right lateral abdomen. Laparoscopic exploration confirmed the reduction of the herniation and identified a defect between the colostomy limb and the abdominal wall. As the remaining sigmoid colon was short and mobilization of the splenic flexure would have been required for colostomy reconstruction through an extraperitoneal route, anterior gastropexy with a technical modification was selected instead of colostomy revision. To preserve the option of future colostomy closure, additional manipulation around the colostomy limb was avoided. To improve gastric mobility and facilitate cranial gastropexy, the left side of the greater omentum was divided (**Fig. 2A**). Using 4-0 nonabsorbable barbed sutures, the anterior wall of the prepyloric antrum was fixed cranially to the round ligament of the liver with 4 stitches (**Fig. 2B**). Subsequently, to straighten the gastric axis and prevent caudal migration, the gastric body was fixed to the upper abdominal wall with 4 additional sutures of the same material (**Fig. 2C**).

**Fig. 2:**
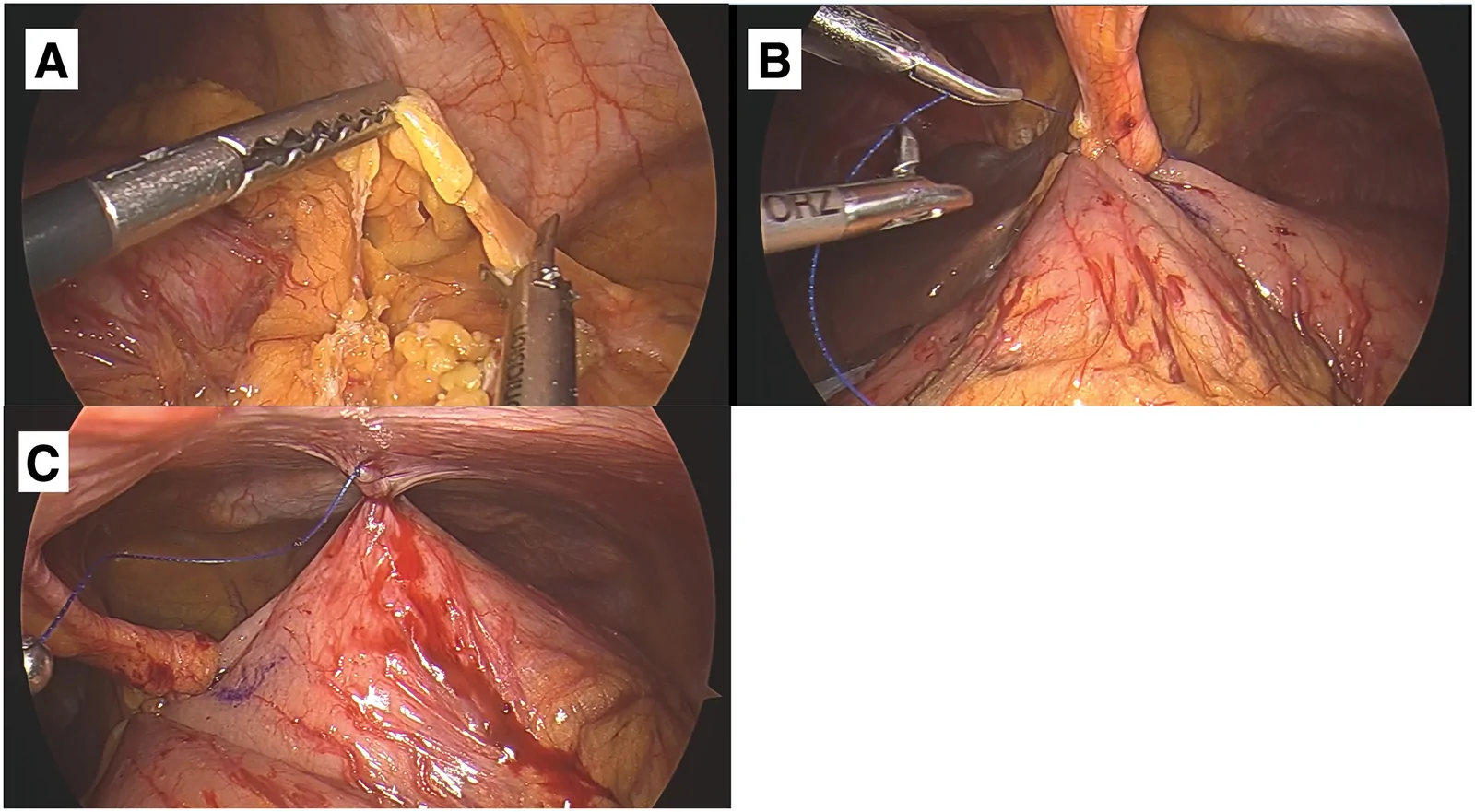
Intraoperative laparoscopic findings. (**A**) The left side of the greater omentum was divided using an ultrasonic scalpel to release traction between the stomach and transverse colon. (**B**) The anterior wall of the prepyloric antrum was fixed cranially to the round ligament of the liver with 4 sutures. (**C**) The gastric body was fixed to the upper abdominal wall with 4 additional sutures.

The postoperative course was uneventful. The patient subsequently received systemic chemotherapy, followed by para-aortic lymph node dissection. No adhesiolysis involving the stomach or gastropexy site was required during the subsequent surgery (**Fig. 3**). Because additional chemotherapy was considered if substantial residual tumor was found in the resected para-aortic lymph nodes, colostomy closure was planned as a staged procedure to avoid delaying postoperative chemotherapy because of potential complications such as anastomotic leakage. Therefore, no additional manipulation around the colostomy limb was performed, and neither colostomy revision nor fixation of the colostomy limb was performed; the lateral defect was left unrepaired. However, pathological examination of the resected lymph nodes revealed only scar tissue without residual cancer, and additional chemotherapy was therefore not administered. Colostomy closure was subsequently recommended; however, the patient declined additional surgery despite repeated recommendations. No recurrence of the internal hernia was observed during 24 months of follow-up, and no evidence of cancer recurrence was detected. Should the patient wish to undergo colostomy closure in the future, the procedure remains technically feasible.

**Fig. 3:**
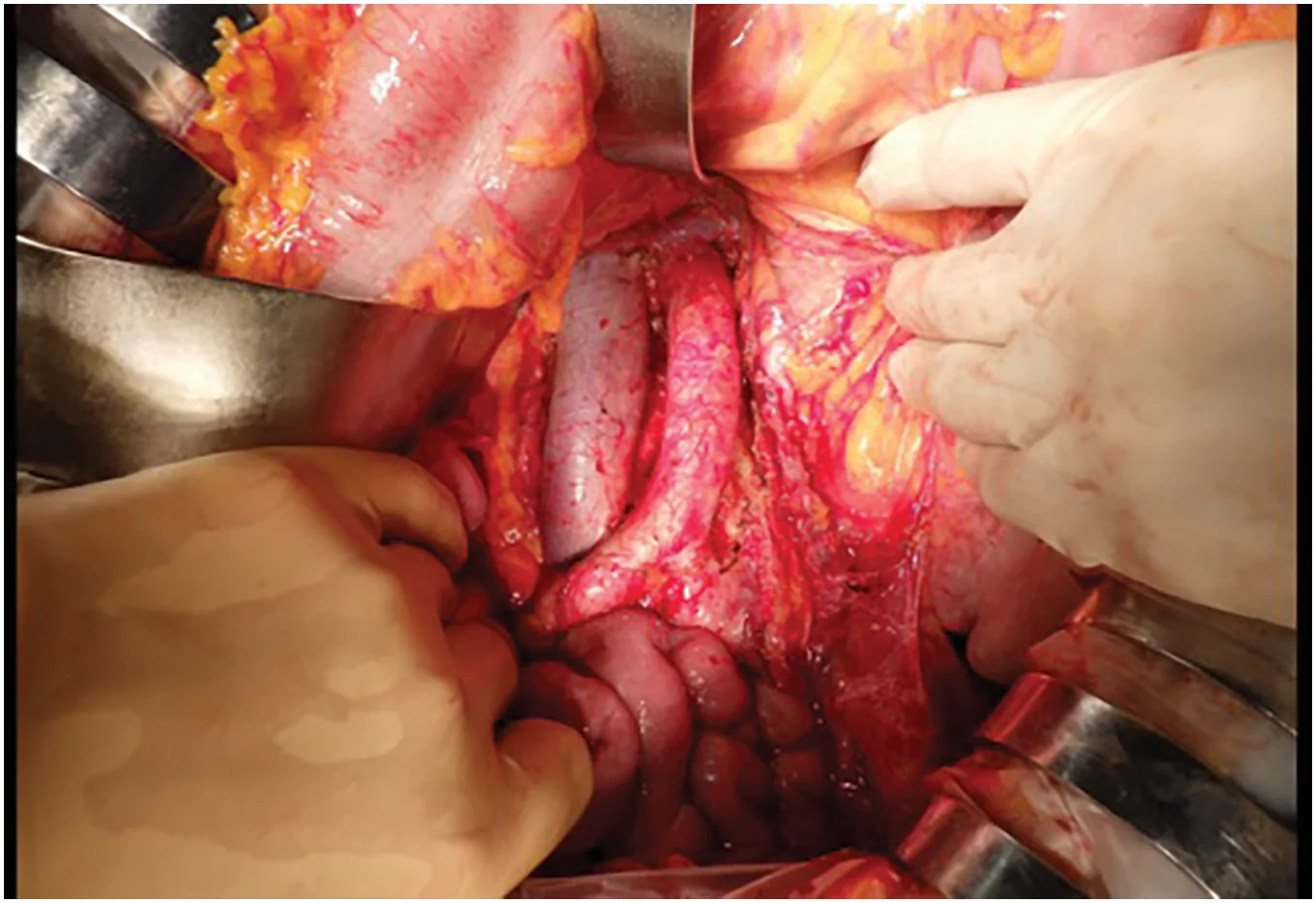
Intraoperative view during para-aortic lymph node dissection. No adhesiolysis was required around the cranial gastropexy site.

## DISCUSSION

Internal hernia involving the stomach after sigmoid colostomy is extremely rare, with only 4 reported cases describing surgical treatment (**Table 1**).^1–4)^ Reported cases have occurred after both laparoscopic abdominoperineal resection and Hartmann’s procedure, with the interval between the initial surgery and the onset of hernia ranging from POD 9 to 11 months. Due to its rarity, no standardized management strategy has been established.

**Table 1:** Reported cases of internal hernia involving the stomach after sigmoid colostomy

Author	Age/sex	Primary surgery	Interval	Surgical approach	Definitive surgery	Follow-up
Shinohara et al.^1)^	81 yr/F	Lap APR	3 mo	Conservative→Elective lap surgery	Colostomy reconstruction with retroperitonealization	20 mo (NR)
Hashida et al.^2)^	79 yr/F	Lap APR	6 mo	Emergency laparoscopy	Colostomy fixation to peritoneum	N/A
Tada et al.^3)^	67 yr/F	Sigmoid colostomy	1 mo (initial), 3 mo (recurrence)	Conservative→Elective lap surgery	Periumbilical gastropexy	2 mo (NR)
Takahashi et al.^4)^	59 yr/M	Lap Hartmann	11 mo	Conservative→ Elective endoscopic surgery	Percutaneous endoscopic gastropexy	4 mo (NR)
Present case	76 yr/F	Lap Hartmann	POD 9	Conservative→Elective lap surgery	Round ligament gastropexy	24 mo (NR)

APR, abdominoperineal resection; F, female; Lap, laparoscopic; M, male; mo, months; N/A, not available; NR, no recurrence; yr, years

CT plays a crucial role in the diagnosis. Coronal images demonstrating a markedly dilated stomach wrapped around the colostomy limb are characteristic. Although nasogastric decompression may temporarily relieve the incarceration, definitive surgical intervention is required to prevent recurrence.

When immediate colostomy closure is not appropriate, 3 surgical strategies have been described in the literature:

(1) reconstruction of the colostomy with retroperitonealization;
(2) fixation of the colostomy limb to the abdominal wall or peritoneum;
(3) gastropexy to restrict gastric mobility.

The first and second approaches eliminate the hernia defect and may prevent the incarceration of other organs, such as the small intestine.^5–7)^ Accordingly, colostomy closure or the first and second approaches would generally be preferred as definitive treatment. Reconstruction with retroperitonealization requires sufficient colonic length and may necessitate mobilization of the splenic flexure. Fixation of the colostomy limb requires direct manipulation of the sigmoid colon and may be more suitable for patients undergoing abdominoperineal resection for rectal cancer with a permanent colostomy, in whom future colostomy closure is not anticipated.^1,2)^ In contrast, immediate colostomy closure was not considered appropriate in the present case because the patient had recently undergone Hartmann’s procedure for obstructive sigmoid colon cancer complicated by obstructive colitis. Furthermore, fixation of the colostomy limb could have increased adhesions around the future anastomotic site or further limited the available colonic length. Therefore, we selected gastropexy to prevent recurrent gastric incarceration while avoiding additional manipulation around the colostomy limb.

Gastropexy does not eliminate the defect itself but instead prevents migration of the stomach into the potential space. It is technically simple and less invasive, particularly when additional manipulation of the colostomy limb is undesirable, such as in patients for whom future colostomy closure remains a potential option. Recently, Takahashi et al. reported successful management of the same condition using percutaneous endoscopic gastropexy under local anesthesia, representing an even less invasive treatment option and a potentially suitable approach for patients who are poor candidates for general anesthesia.^4)^ Tada et al. reported laparoscopic anterior gastropexy in which the prepyloric antrum was fixed to the periumbilical region (**Fig. 4A**).^3)^

**Fig. 4:**
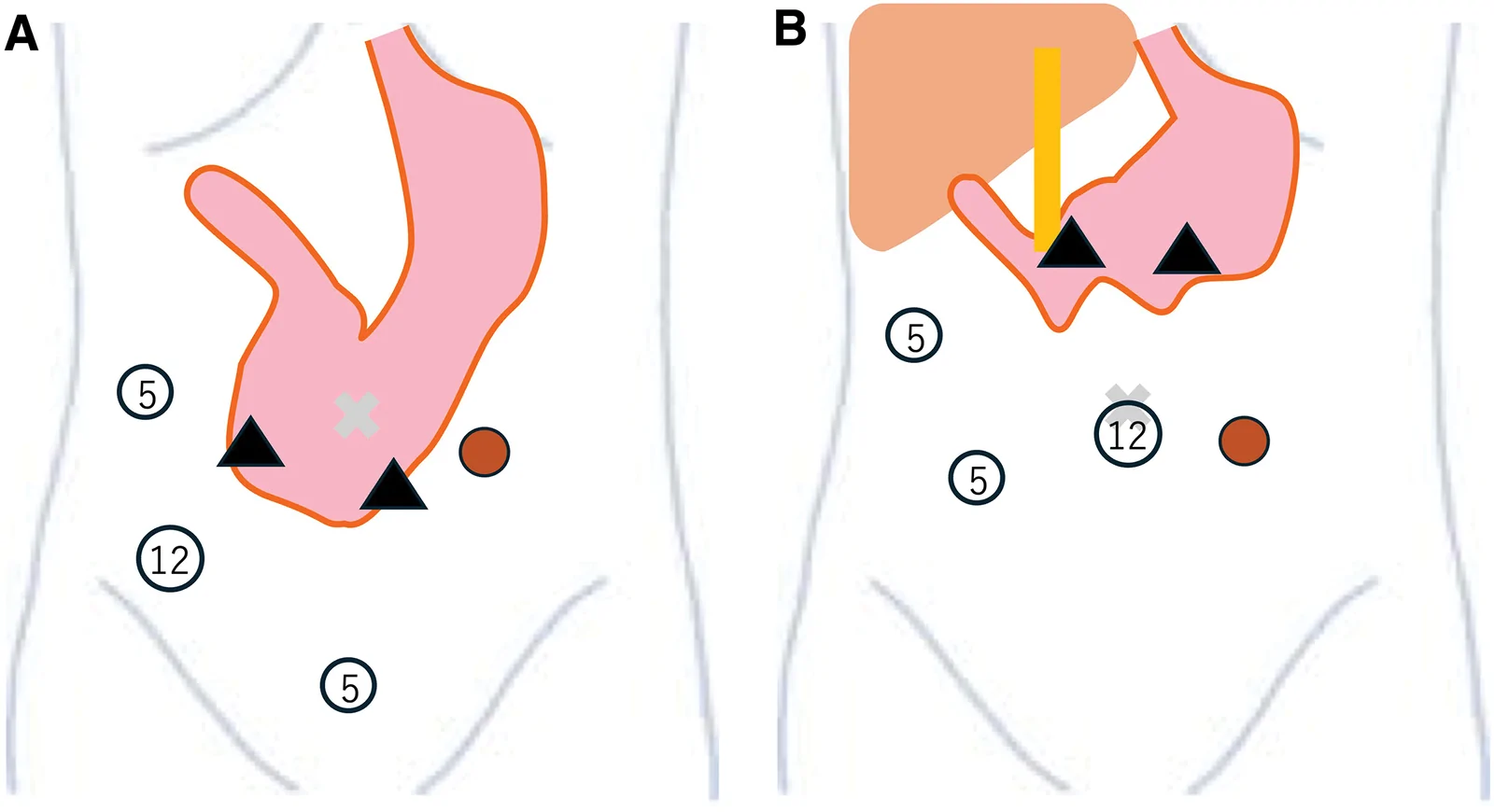
Gastropexy techniques. Black triangles indicate the suture sites, and black circles indicate the trocar sites. The brown circle indicates the stoma. (**A**) In the case reported by Tada et al.,^3)^ the prepyloric antrum was fixed in the periumbilical region using a Funada-style loop gastropexy device. (**B**) In the present case, the prepyloric antrum was fixed to the round ligament and the gastric body to the upper abdominal wall using two 4-0 nonabsorbable barbed sutures, with 4 stitches placed at each fixation site.

The present procedure should be regarded as a technical modification of conventional laparoscopic anterior gastropexy rather than a novel surgical strategy. In the present case, we selected the round ligament as the cranial anchoring point based on 2 anatomical considerations (**Fig. 4B**). First, unlike direct fixation of the prepyloric antrum to the anterior abdominal wall, fixation to the round ligament places the prepyloric antrum between the anterior abdominal wall and the retroperitoneally fixed duodenum. We considered that this anatomical relationship might help preserve a more physiological gastroduodenal alignment and avoid excessive ventral angulation of the gastric outlet. Second, the round ligament, a fibrous remnant of the obliterated umbilical vein, may provide a stable anchoring point. These potential advantages remain theoretical because they were not objectively evaluated in the present case. Furthermore, because multiple procedures, including division of the greater omentum, fixation of both the prepyloric antrum and gastric body, and multipoint suturing, were performed simultaneously, the individual contribution of round ligament fixation cannot be determined, and its superiority over conventional parietal fixation cannot be established.

In addition to these anatomical considerations, no adhesiolysis involving the stomach or gastropexy site was required during the subsequent para-aortic lymph node dissection. However, this postoperative finding does not demonstrate a specific advantage of round ligament fixation over previously reported gastropexy techniques. The hernia defect should generally be addressed to prevent recurrent internal herniation; however, when future colostomy closure is anticipated and manipulation around the colostomy limb is undesirable, gastropexy may be a feasible alternative. Leaving the hernia defect unrepaired may theoretically allow future internal herniation involving other abdominal viscera, and this potential risk should be considered when gastropexy is selected without direct repair of the defect. Taken together, the present technical modification may be a feasible option in selected patients requiring gastropexy, potentially maintaining physiological gastroduodenal alignment and avoiding excessive angulation of the gastric outlet.

## CONCLUSIONS

In conclusion, internal hernia involving the stomach after sigmoid colostomy is extremely rare, and its optimal management remains unclear. Laparoscopic anterior gastropexy with a technical modification using the round ligament may represent a feasible and minimally invasive option for preventing recurrent gastric incarceration in selected patients, potentially maintaining physiological gastroduodenal alignment and avoiding excessive angulation of the gastric outlet.

## DECLARATIONS

### Availability of data and materials

The datasets supporting the conclusions of this study are included in this article and its additional files.
